# Sex Steroids as Regulators of Gestural Communication

**DOI:** 10.1210/endocr/bqaa064

**Published:** 2020-04-20

**Authors:** Daniel J Tobiansky, Matthew J Fuxjager

**Affiliations:** Department of Ecology and Evolutionary Biology, Brown University, Providence, Rhode Island

## Abstract

Gestural communication is ubiquitous throughout the animal kingdom, occurring in species that range from humans to arthropods. Individuals produce gestural signals when their nervous system triggers the production of limb and body movement, which in turn functions to help mediate communication between or among individuals. Like many stereotyped motor patterns, the probability of a gestural display in a given social context can be modulated by sex steroid hormones. Here, we review how steroid hormones mediate the neural mechanisms that underly gestural communication in humans and nonhumans alike. This is a growing area of research, and thus we explore how sex steroids mediate brain areas involved in language production, social behavior, and motor performance. We also examine the way that sex steroids can regulate behavioral output by acting in the periphery via skeletal muscle. Altogether, we outline a new avenue of behavioral endocrinology research that aims to uncover the hormonal basis for one of the most common modes of communication among animals on Earth.

Gesture plays a fundamental role in animal communication. As humans, we are intimately aware of this fact, given that individuals from all cultures across the globe use gesture to convey ideas, thoughts, and feelings to others (Fig. 1A; (1)). It is therefore unsurprising that linguists and psychologists have spent decades creating a lexicon to classify the various modes of gestural communication, as well as a framework to understand how each of these modes likely function (2-4). Building on this work is a growing body of mechanistic studies, which probe how the brain and body might control gestural communication. Much of this research suggests that the neural substrates responsible for mediating the perception and production of speech also mediate the gesticulation—voluntary or not—that so often accompanies our everyday conversations (5-7). In many ways, this work only scratches the surface of the intricate mechanisms that underlie gestural communication.

**Figure 1.**
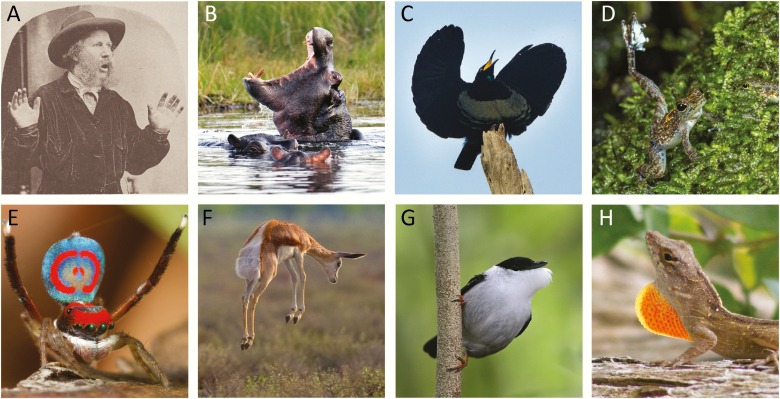
Examples of gestures in vertebrates. (A) A gesture associated with surprise in humans. This photograph was used by Charles Darwin in his book, *The Expression of the Emotions in Man and Animals* [(114); licensed under Getty Museum Open Content Program]. (B) A territorial gesture of hippopotamus [*Hippopotamus amphibious* (115); credit: Robert A. Tobiansky, with permission]. (C) A mating gesture from a male Victoria’s riflebird (*Ptiloris victoriae*), whereby he hold out his wings and move his head side-to-side (31) (credit: Francesco Veronesi licensed under CC BY-SA 2.0). (D) A Bornean rock frog (*Staurois parvus*) producing a foot-flagging gesture to compete with rival males at a breeding site [(116); credit: Vienna Zoo/Doris Preininger, with permission]. (E) A male peacock spider (*Maratus splendens*) performing a mating display in which it raises its abdomen and waves its hind legs [(117); credit: Jurgen Otto licensed under CC BY-NC-ND 2.0]. (F) A springbok (*Antidorcas marsupialis*) performing a stotting display, in which it leaps into the air to notify predators that they have been detected [ (118); credit: Yathin sk licensed under CC BY-SA 3.0]). (G) A male white-collared manakin (*Manacus manacus*) in a “beard up” posture, which typically occurs when the male is dancing for the female [ (65); credit: Steve Garvie licensed under CC BY-NC-SA 2.0]. (H) A male brown anole (*Anolis sagrei*) gestures to conspecifics and potential predators by extending its dewlap (orange throat patch) in a rhythmic pattern [(119) credit: touterse, licensed under CC BY 2.0].

In this mini-review, we explore this topic from the point of view of the endocrine system, assessing how hormones modulate the integration of body movement into animal communication programs. We define gesture as the process by which an individual actively moves its face, limbs, and/or body in a coordinated manner (and often with speech of vocalization) to help facilitate communication (8). This broad definition not only encompasses many of the functional subclassifications of gesture (eg, sign language, pantomime, co-speech gesture), but also recognizes the fact that gestural communication in found across the entire animal kingdom. In this spirit, we begin our review by focusing on neural and neuroendocrine regulation of co-speech gesture in humans. We then expand our discussion beyond humans and give an overview of the hormonal control of gesture in a wider range of taxa. This comparative approach reveals the diverse ways in which hormone systems are involved in the physiological coordination of signaling with the body alongside sound production or, in some cases, without it.

## Sex steroids and communication

Why would hormones influence animal communication, and which hormones are most likely to do so? The answer to the first question centers around the major role of hormone action, which provides context-appropriate regulation of behavior (9, 10). In this way, it makes sense that hormones would also regulate the systems controlling communication because this would ensure that language and/or other signals are produced at the right time and place. The answer to the second question requires an understanding of the function of communication. In this brief review, we focus on communication used to navigate sexual encounters, with the aim of increasing reproductive fitness. For these interactions, we can reasonably expect that hormones underlying sexual behavior modulate communication.

This brings us to sex steroids—namely, androgens, estrogens, and progestins. These hormones are principally produced by the gonads, and mediate sperm production in males and follicular development in females (9). At the same time, these hormones are also released into the bloodstream, where they circulate throughout the body and act on target tissues that express androgen receptors, estrogen receptors, and progesterone receptors. Here, sex steroids act to help facilitate the organization of the reproductive phenotype (eg, development of the genitals, reproductive tract, secondary sexual characteristics, and neural circuits underlying sexual behavior), as well as the regulation of sexual behavior during adulthood. This collectively means that the same hormones regulate both the expression of sexual traits (morphological and behavioral) and the production of mature gametes. Such a design is by no means an evolutionary accident—rather, this coupling is thought to be adaptive, ensuring that reproductive systems are expressed precisely when gametes are available (10).

Because gestural communication often facilitates reproduction, it stands to reason that sex steroids regulate its production in this context. But, how? We know surprisingly little about this topic (11). One factor that makes the question especially interesting is that gestural communication also has nonreproductive functions. This raises the possibility that, in addition to mediating the performance of sexual gestures, sex steroids also help govern how and/or when animals switch between producing sexual gestures and nonsexual ones.

## Regulation of gestural communication in humans by sex steroids

**Figure 2.**
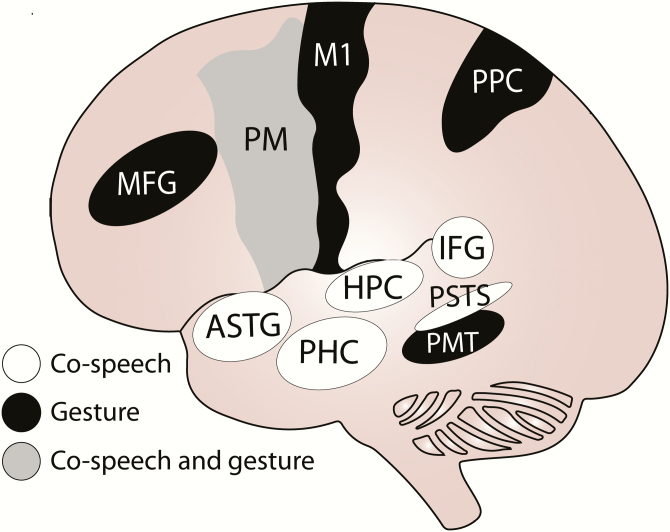
Neural nodes associated with co-speech gesture and other forms of gesture (eg, pantomime) in humans. Neural nodes associated with co-speech gesture (white or gray) include the premotor cortex (PM), hippocampus (HPC), parahippocampal cortex (PHC), left inferior frontal gyrus (IFG), anterior superior temporal gyrus, (ASTG), and bilateral posterior superior temporal sulcus (PSTS). Neural associated with other forms of gesture (eg, pantomime) (black) include the PM, areas of the middle frontal gyrus (MFG), posterior parietal cortex (PPC), the primary motor cortex (M1), and the posterior medial temporal cortex (PMT). The PM is shaded gray to signify its role in both co-speech and gesture.

The brain controls the production of gestural communication, determining not only when it is performed, but also how it is performed. Some of the most interesting work that explores this process is in humans using functional magnetic resonance imaging to determine brain regions associated with different forms of gestural communication. For example, studies in men and women investigating the neural basis of co-speech gestures—the hand movements we tend to make while we speak (12, 13)—find that hand movements during speech are correlated specifically with indices of increased activity in brain areas otherwise linked with language production (Fig. 2) (7, 14). This includes the left inferior frontal gyrus (IFG, or Broca’s area), anterior superior temporal gyrus, bilateral posterior superior temporal sulcus, left hippocampus, parahippocampal cortex, and ventral and dorsal premotor areas. These brain regions are involved in word retrieval and speech articulation, as well as motor control of the hands (15, 16). At the same time, co-speech gesture is also associated with increased activity in brain areas that make up the gesture network, which governs pantomime gestures, imitating gestures, and tool use (6, 17). Regions include the premotor and primary motor, left posterior parietal, posterior middle temporal, and middle frontal areas (Fig. 2). Overall, these data point to the existence of gesture and language networks that share a variety of nuclei.

Even less is understood about how sex steroids act on these areas to modulate co-speech gesture. The first hint that such effects may occur comes from work in humans and other nonhuman animals showing that many of the brain regions mentioned above either express steroid hormone receptors (estrogen, androgen, or progesterone receptors) or are connected to brain areas that have these receptors (18-23). The second hint comes from a small number of studies that suggest sex steroids act in the central nervous system to influence language production (and perception), as well as motor control. For instance, neuroimaging studies in adult men and women reveal that gray matter volume in the left IFG is positively associated with levels of circulating 17β-estradiol (the most bioactive estrogen), but negatively associated with circulating testosterone (24). This suggests that estrogens and androgens are capable of acting in these parts of the brain to induce morphological changes, although the time at which these effects occur (either during development or in adulthood) remains unclear. Other work points to strong organizational effects of sex steroid action on brain regions that underlie language production, as both pre- and postnatal effects of steroids influence language development skills later in life (25, 26). Interestingly, these effects would suggest a manifestation of sex differences in co-speech gesture, yet this does not seem to be the case (eg, (2)).

Sex steroids similarly impact motor centers in the brain, which are also involved in the production of co-speech gesture. Preliminary research, for example, suggests that cancer patients undergoing androgen deprivation therapy exhibit a marked decline in gray matter within the primary motor cortex (27). Moreover, patients with congenital adrenal hyperplasia, a condition in which an enzyme deficiency leads to the overproduction of adrenal androgens, show enhanced performance on gross motor and visuomotor tasks, but diminished performance of fine motor skills (28). These effects may be rooted in sex steroid-dependent regulation of motor cortical connectivity during development (29). However, these effects are difficult to reconcile, and thus suggest that regulation of motor command by sex steroid action is a complex process that varies by brain region and across developmental stages.

Although this small body of research implies that sex steroids may exert some influence on the neural basis of co-speech gesture, we must recognize that exceptionally little is known about how these effects are borne out. The first step in addressing this gap is to generate a better understanding of how the brain controls co-speech gesture. This is admittedly a tall order, but ultimately a fascinating question given the degree to which such behavior in ingrained in our communication [even congenitally blind patients produce co-speech gesture (3)]. The next step is to understand how and where sex steroids might act in the brain to modulate this process. As reflected in some of the research cited above, studies in congenital adrenal hyperplasia patients are an obvious place to begin.

## Beyond humans: Insights from gestural communication in vertebrates

One might expect that the study of gestural communication in nonhuman animals would quickly dry up. How many species communicate with gesture, and what role could hormones possible play in this process? The answer to the first part of the question is simple: Nearly all social species use gesture to signal to others, whether they are of the same or different species. Gestures vary tremendously among taxa, ranging from chest pounding to dancing behavior (Fig. 1) (30-36). Often, these displays mediate courtship of potential mates or competitive behavior between rival males (or both). The answer to the second question about hormone regulation of these displays is similarly straightforward: Sex steroids are vital to the production of gestural signals.

## Modulation of gesture via sex steroids in the central nervous system

Our understanding of how sex steroids regulate gestural displays starts with classic studies that probe the mechanisms underlying postural and reflexive control necessary for copulation and courtship. One of the best examples comes from work on lordosis in rats. This is not a gesture per se; rather, it is a stereotyped mating posture that signifies sexual receptivity and thus mediates successful mating (37). Nonetheless, studies of lordosis behavior in female rodents serve as a template for understanding how hormone systems interact with the brain to influence motor control, which in turn informs our broader understanding of neuroendocrine regulation of gesture. Decades of research shows that a central node in the activation of lordosis is the ventromedial nucleus of the hypothalamus (VMH) (38, 39). Cells in this nucleus project to a premotor area called the periaqueductal gray (PAG) of the midbrain, which in turn projects to the nucleus retroambiguus (NRA) in the caudal medulla. The NRA sends several bulbospinal projections to spinal motoneurons that innervate much of the body (40). To activate lordosis, estrogens must first prime the VMH by acting through both canonical and noncanonical pathways (37). Subsequent exposure to estrogens and progestins then increases the probability that lordosis occurs in response to sensory input from the female’s hind flanks (ie, sensory input from a mounting male) and olfactory information from the vomeronasal organ. Additionally, sex steroid action in these same brain areas also likely mediates a sexually proceptive behavior in female rats known as ear wiggling, which falls well within the definition of a gesture. This behavior signifies willingness to mate to the male and is similarly sensitive to estrogen and progestin priming (41), but far less is known about its underlying neural basis. Nonetheless, this work collectively shows the critical role that sex steroid action plays in setting the neurological stage for refined motor command. Indeed, without estrogen priming and subsequent estrogen/progestin action, lordosis behavior and proceptive gestures fail to manifest (39).

In males, the medial preoptic area (mPOA) is the principal node that mediates sexual behavior, as opposed to the VMH (42, 43). More recent work in birds nicely illustrates how the mPOA (often called the POM in birds) is similarly linked to the systems that govern motor control, given that the mPOA itself does not connect to the spinal cord. Rather, the mPOA projects to the intercollicular nucleus (ICo), a complex of nuclei homologous to the mammalian PAG (44). The cells that receive input from the mPOA then fire on a subnucleus within this complex called the dorsomedial nucleus (DM) (45). To biologists studying display behavior, the DM is an especially interesting part of the brain because its electrical stimulation immediately causes birds (even ones who are anesthetized) to produce vocalizations used for social communication (46). Neuroanatomical work indicates that the DM sends projections to 2 other important areas, the vocal motor nucleus (nXIIts) and the nucleus retroambigualis (RAm) (47). RAm, which is homologous to the mammalian NRA, projects to several spinal motoneurons that innervate thoracic and lumbar expiratory muscles, as well as major cloacal muscles that actuate copulatory reflex movements (48). When testosterone acts via the mPOA, it stimulates the production of different cloacal responses. In male quail, for instance, testosterone modulation of the mPOA significantly increases the number of rhythmic cloacal sphincter movements in response to a female’s presence (49). As such, we again see how central sex steroid action modulates specific movement programs involved in mediating sexual interactions.

**Figure 3.**
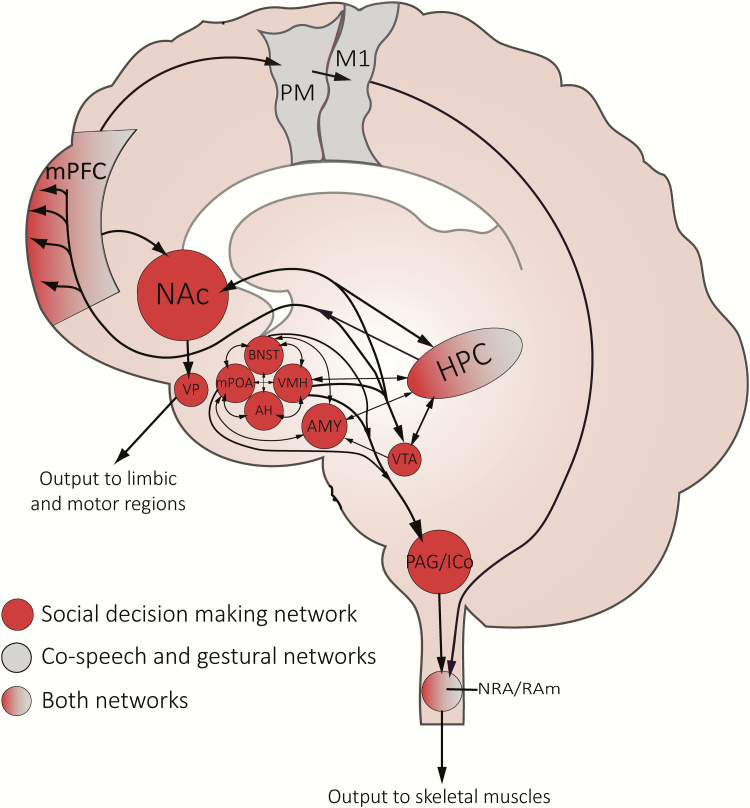
Regions of evolutionary conserved social decision-making network and its interaction with the gesture/co-speech gesture network in the human brain. Select nodes of the social decision making network (red), which include areas from the social behavior network and the motivational/reward circuit (described by 50). Regions involved in co-speech gesture and other forms of gesture (eg, pantomime) are shaded in gray. Nodes that act as an interface between the social decision making network and co-speech/gestural networks a shaded with a gray-red gradient (ie, “Both networks”). Abbreviations: AH, anterior hypothalamus; AMY, amygdala; BNST, bed nucleus of the stria terminalis, HPC, hippocampus; ICo, intercollicular nucleus; M1, primary motor cortex; mPFC, medial prefrontal cortex; mPOA, medial preoptic area; NAc, nucleus accumbens; NRA, nucleus retroambiguus; PAG, periaqueductal gray; RAm, nucleus retroambigualis; VMH, ventromedial hypothalamus; VP, ventral pallidum; VTA, ventral tegmental area.

As the body of work described above charts out how sex steroid action in the brain can influence basic motor control for sex, we suspect that similar processes underlie the production of gestures used to mediate courtship and male–male competition. In this case, however, regulation of gesture is likely rooted in the functionality of the social decision-making network, which is a group of interconnected nuclei that collectively govern social behavior (Fig. 3) (50, 51). All nodes in this circuit are sensitive to sex steroids, including androgens, estrogens, and progestins. The network itself includes the VMH, mPOA, and several other hypothalamic nuclei that help control aspects of motivation, arousal, reward, and reinforcement. Importantly, in this list of neural loci is the PAG/ICo, which is thought to serve as the major interface to downstream motoneuron processes. Relative activity of these nuclei helps determine an animal’s social output, while steroid hormone action directly mediates activational patterning (52). Importantly, social experience can alter the sensitivity of these brain regions to steroid hormone action, and thus predict meaningful changes in future behavioral interactions (53). Some of the most compelling work to suggest that nodes of the social decision-making network influence gesture and/or posture comes from studies in songbirds (Order: Passeriformes). For example, work in canaries (*Serinus canaria*) shows that a nucleus within the song control system—the robust nucleus of the arcopallium (RA, a telencephalic nucleus)—directly innervates both the DM (part of the ICo) and RAm (between which there are also connections; see earlier) (54). Integration between these 2 networks is thought to underlie the functional coupling of social display and other relevant behavior, such as motor control of the cloaca and copulation solicitation displays. More importantly, this work provides a template to envision how the nervous systems can integrate aspects of motor control for different types of behavior (eg, song and postural or gestural displays) in a context-appropriate manner.

This research in canaries is important for a few other reasons. First, it again highlights the significance of the hindbrain nuclei (NRA/RAm) for motor skills involved in sexual display. The nuclei are typically viewed as respiratory premotor nuclei, but they are also involved in the execution of other tasks that require alteration of respiratory patterning such as vomiting and abdominal straining (55). This, alongside the fact that NRA/RAm are involved in sexual postures (40, 56), has led others to argue that both nuclei play a broad role in motor control by operating as behavioral pattern generators with numerous connections throughout the nervous system (55). Inputs to NRA/RAm, like the VMH, are sensitive to sex steroids, and studies suggest that estrogenic action within these inputs can trigger neuronal outgrowth of axons from the NRA/RAm down into the spinal cord (57, 58). Thus, sex steroids appear capable of acting centrally to modulate how the brain can send commands to various parts of the spinal cord. Research in male ruffed grouse (*Bonasa umbellus*) provides a potential link between these effects and gestural display. Males of this species perform a territorial and courtship display, in which they stand erect on a log in the forest and rapidly flap their wings up and down to generate a low-frequency drumming noise that booms through the forest (59). Histological studies suggest that this behavior is associated with greater expression of aromatase in certain regions of the VMH (60); thus, enhanced estrogenic production and action in this nucleus may augment NRA/RAm connectivity with spinal interneurons and motoneurons.

Second, the work with canaries also suggests that other brain areas outside the social decision-making network likely contribute to display behavior, and thus gestural displays. The RA is part of the avian arcopallium, which has clear premotor functions. For example, studies show that wing and leg movements are associated with increased expression of immediately early genes in certain arcopallial nuclei (61), while other work indicates that parts of the arcopallium project to the pontomedullary reticular formation in the brainstem (62, 63). If this latter area is electrically stimulated then basic locomotory patterns (eg, walking, wing flapping) are evoked (64). Research also provides intriguing support for the idea that these pathways are under androgenic modulation in species that perform elaborate gestural displays. Golden-collared manakins (*Manacus vitellinus)*, for example, express high levels of androgen receptor throughout much of the arcopallium. These tropical birds produce a courtship display in which they rapidly jump around the forest floor, snapping their wings together in mid-air to generate a loud, firecracker-like pop (65). Avian species that do not produce these displays express little to no androgen receptor in the arcopallium (66), suggesting that increased androgenic sensitivity within this brain region is related to the bird’s unusual display routine. Interestingly, one of the species that does not express androgen receptor in the arcopallium is the ruffed grouse (see earlier (60)); thus, androgenic action in the arcopallium itself is not always associated with gesture.

Another important level of the nervous system to consider is the spinal cord. Motoneurons innervating the wing muscles of golden-collared manakins express high levels of androgen receptor, particularly when compared to other birds that do not produce gestural displays with their wings (67). This suggests that androgens help mediate display behavior by acting directly on the motoneurons that relay information from the central nervous system to the muscle itself. Similarly, work in the Japanese quail (*Coturnix japonica*) shows that the motoneurons innervating the cloacal muscles contain high levels of estrogen receptor, which stands in contrast to the dearth of estrogen receptor in most other regions of the spinal cord’s ventral horns (68). At the same time, the dorsal horns of the bird’s spinal cord make their own estrogens, likely feeding the receptor population in the cloacal motoneurons (69). This suggests that estrogens may act locally within the lower spinal cord to help mediate motor control of the cloaca during sexual interactions.

Additional research suggests that sex steroids are important regulators of neural functioning within the spinal cord. Spinal interneurons, for instance, express estrogen and androgen receptor (70-72). These cells are critical to the control of muscle synergies, which represent the independent, modular movement programs that make up complex behavioral output (73, 74). Thus, if sex steroids regulate the morphology and connectivity of spinal interneurons either during development or in adulthood, they may have a profound effect on the ontogeny and/or activation of gestural displays. There is a deep and rich literature that explores the neural basis of muscle synergies and their implications for behavioral control, but this work is seldom addressed in the field of behavioral endocrinology, and our understanding of steroidal control of gestures could benefit greatly from incorporating these ideas.

Considering the work described above, it is clear that we still know little about how the brain controls animal gestural displays, or how sex steroids act on the brain to modulate this behavior. Studies are needed to pinpoint specific brain regions or neural circuits associated with gestural control, independent of other phenomena (eg, vocalizing, locomotion). From here, we can begin to assess how actions of estrogen, androgens, and progestins modulate these brain nuclei to influence gesture. Other intriguing questions will undoubtedly emerge from studies in this area, such as how central steroid action influences the development of gestural displays (75). Might such effects account for sex differences in gestural behavior? Indeed, future research that embraces this approach promises to redefine our thinking about adaptive motor command and how its mechanisms are embedded in the central networks that underlie the basic elements of social behavior—sex, courtship, territoriality, and cooperation.

## Steroidal modulation of gestures via the periphery

We must also recognize that sex steroids act peripherally to influence gestural communication. This process can be conceptualized as steroidal modulation of substrates that respond to “instructions” sent from the brain about how to move the body in a specific manner. The idea makes more sense if we consider the nature of the gestures that many animals produce for communication, especially those that demand performance skills and abilities that would not normally be attributed to the species in question (eg, bouts of extreme speed, strength, endurance, or any combination thereof) (76). So how might this work? There are 2 major ways for sex steroids to peripherally regulate the motor systems underlying animal performance.

First, sex steroids might act directly on the musculoskeletal system to adjust how the striated muscle and its associated structures actuate movement. In particular, androgenic steroids, like testosterone, play a powerful role in the regulation of muscle, which expresses high levels of androgen receptor compared with many other tissues in the body (77, 78). Androgens, therefore, induce a variety of effects on muscle fibers, increasing their size, fiber type composition, and ability to handle calcium ions (79-83). Estrogens and progestins are also known to act on muscle, although their effects are much less clear. Some research suggests that these 2 hormones mediate aspects of muscle performance, like endurance (84, 85). At the same time, other work implies that estrogenic hormones help buffer muscle tissues from use-related damage by directly mediating myocytic repair (86).

Research that directly ties gestural displays to sex steroid action at the level of muscle focuses specifically on androgenic systems. For example, in a fish called the blue-banded goby (*Lythrypnus dalli*), males defend nest sites and court females by performing swim displays, in which they repeatedly extend and retract their pelvic and dorsal fins. The muscles that actuate these fin movements contain high levels of androgen receptor compared with other major muscles that power swimming (87). Moreover, levels of androgen receptor in these same fin muscles are positively associated with the rate at which individuals perform their display, suggesting that androgenic regulation augments muscular control of fin movements related to sexual signaling. Additionally, studies in golden-collared manakins (the acrobatic displaying bird mentioned earlier) shows that activation of androgen receptor in the wing musculature is necessary for most complex gestural signals. If androgen receptors in these tissues are blocked by a peripherally selective androgen receptor antagonist, then males produce fewer gestural displays and slow down the signals they do broadcast to females (88). These effects have been traced back to the muscle that actuates the bird’s rapid wing movements, on which androgens act to increase twitch speeds to ‘superfast’ levels (89). These effects are also observed at the molecular level. For instance, in manakins, androgens upregulate the transcription (messenger ribonucleic acid) of both parvalbumin, a myocytic calcium buffer, and insulin-like growth factor 1, a growth factor that stimulates muscle growth (80). Studies in other taxa similarly show that androgens completely remodel the transcriptomic profile of muscle in a way that undoubtedly affects multiple aspects of performance, such as speed, endurance, force production. (90, 91). Comparative work in birds even shows that the impact of androgens on muscle can vary not only among different tissues, but also across different species (92).

Of course, the role of androgen–muscle interactions in behavior is much more complex than stated above. We would argue that the literature is beginning to assess how androgens help reorient organismal behavior within larger, multifaceted performance landscapes, rather than altering singular aspects of speed, strength, endurance, etc. This idea is anchored by studies showing that most performance elements are tied together in complex trade-off schema (93-95). Speed, for example, often comes at a cost to force production and the resistance to fatigue (96). In this way, androgens might act on muscle to balance it along this trade-off. In golden-collared manakins, for example, androgenic action not only increases the speed of display muscle, but also likely induces its hypertrophy (89). The latter effects may therefore recoup strength needed to power locomotion, which is otherwise depleted by the substantial increase in speed. If so, then androgens are acting as modulators of performance trade-offs, thereby helping individuals mitigate putative locomotory costs that would accrue as a result of an evolutionary push for an effective (eg, fast) display.

The second way that peripheral androgenic action might regulate mechanisms of gestural control is by indirect modulation of the nervous system (97, 98). This idea originates from work in male rodents, which shows that androgens act on the musculature of the penis to maintain the morphology of the motoneurons that connect these tissues to the spinal cord (99). This occurs because androgens upregulate neurotropic and growth factors, which retrogradely travel from the periphery to the spinal cord (100, 101). There, these proteins initiate a host of changes to the motoneuron, such as increasing its dendritic arborization and soma size (99, 102). Such features of the motoneuron impact its functionality and capacity for integration (103-105).

Unfortunately, little work has been carried out to explore how peripheral steroid action influences central control of behavior. Recent work in frogs, however, suggests that it likely plays a role. Species like the Bornean rock frog (*Staurois parvus*) have evolved intricate waving displays with their hindlimbs to compliment acoustic communication in especially noisy environments, such as under a waterfall. Androgens help activate these displays likely by acting on the musculature that extends, rotates, and retracts the femur (106). Because these displays are believed to be about the slow execution of a gesture that requires great skill and motor command, it is thought the androgenic regulation of muscle helps maintain a spinal phenotype that supports the ability to produce the signal (ie, foot flagging behavior) (106, 107).

Going forward, there are many gaps to fill with regard to our knowledge about sex steroids and their ability to act via the musculoskeletal system to modulate gestural displays. Hints from endocrinological research that estrogens and progestins modulate muscle performance should be examined in a behavioral context. Are the effects of these hormones on select muscles necessary to generate certain types of display movements? If so, why? How do these hormones change muscle physiology in a functionally meaningful manner? Equally important is a more thorough understanding of how sex steroid-mediated signaling from the muscle to the spinal cord regulates behavior. We have known about this phenomenon for decades, yet it has gained little steam in terms of informing our understanding of behavioral modulation. How might changes to spinal cord morphology induced by peripheral sex steroid action alter motor skills and/or muscle synergies that guide complex movements used to communicate?

## Steroid hormones systems and the evolution of gestural communication

An emergent view from the research described above is that sex steroid systems act as channel through which gestural signals can evolve. Accordingly, selection for specific gestural patterns and routines may proceed through concurrent changes to the systems by which sex steroids regulate motor command (107-109). Support for this idea comes from comparative work that shows a positive relationship between expression of androgen receptor in specific target tissues and species variation in gestural display complexity. In *Anolis* lizards, for example, species that exhibit greater rates of territorial push-up display express higher levels of androgen receptor in their forearm muscles (110). Push-up rate was not related to muscle fiber size or body size, and these effects accounted for the shared evolutionary history among the taxa. Overall, this work points to a clear link between the properties of gestural display and the evolution of the androgenic system in the muscles that actuate it. Research in other taxa point to similar effects (106, 111), including work in tropical birds (112). This latter study is important because, while it shows a positive relationship between levels of androgen receptor transcription and taxonomic variation in gestural display complexity, it also reveals that such coevolutionary relationships do not exist with regard to estrogen receptor. Moreover, this study also looks at androgen and estrogen receptor in the spinal cord, and similarly fails to uncover evidence of a coevolutionary relationship between these transcripts and display variation. Thus, the evolutionary linkage between gesture and sex steroid systems appears to be specific to androgenic signaling and muscle.

Building on this idea is work that explores how the androgen receptor itself might evolve. While steroid receptors are thought to be highly conserved, recent work shows that androgen receptor protein in birds does vary across taxa (113). In particular, there are 2 avian families (Class: Aves) that show substantial deviation in the polarity and hydrophobicity of select androgen receptor domains—manakins (family: Pipridae) and hummingbirds (family: Trochilidae). Many species within these families produce extraordinary gestural displays, pointing to yet another link between the evolution of the androgenic system and elaborate physical displays. Although it is currently unclear how these precise structural changes to the androgen receptor influence its ability to induce signaling, studies of the androgen receptor’s biochemistry imply that such modifications likely impact post-translational regulation of the protein’s functional potency (113). To this end, if sexual selection for gestural displays drives the evolution of these changes to the androgen receptor, we must recognize that this might influence other androgen-dependent processes unrelated to social display.

## Conclusions

In sum, we briefly reviewed the literature that currently provides the basis of our understanding about the relationship between the endocrine system and gestural communication in vertebrates. Most of this work focuses on regulation through sex steroids, acting on pathways in the brain that mediate sexual reflexes and postures. Such research is just beginning to extrapolate these ideas to nonmodel systems, which have evolved highly complex gestural displays that incorporate dance, acrobatics, and other movements requiring a high degree of fine motor control. It appears that the key to understanding these processes lies in untangling the neural pathways that integrate facets of sociality with motor control systems. However, sex steroids also appear to act via the spinal cord and muscular system to prime the body thereby allowing it to appropriately respond to “instructions” from the brain. We end the review with a brief reflection on the evolution of these displays—namely, the idea that changes to the mechanisms underlying steroid action throughout the neuraxis can help precipitate adaptive modifications to gestural communication systems. Thus, sex steroid systems currently appear to be a primary conduit for the evolution of sexual movement programs.

## Abbreviations

DM: dorsomedial nucleus
ICo: intercollicular nucleus
IFG: inferior frontal gyrus
mPOA: medial preoptic area
NRA: nucleus retroambiguus
VMH: ventromedial nucleus of the hypothalamus
PAG: periaqueductal gray
RAm: nucleus retroambigualis

## Additional Information

***Disclosure Summary:*** The authors have nothing to disclose.

***Data Availability:*** Data sharing is not applicable to this article as no datasets were generated or analyzed during the current study.
